# Characterizing Human Visual Performance in Dim Industrial Environments: An Eye-Tracking Sensor-Based Study on Mine Monitoring Interface Layouts

**DOI:** 10.3390/s26134310

**Published:** 2026-07-07

**Authors:** Junqing Hao, Rui Chen, Wei Zong

**Affiliations:** School of Architecture and Design, China University of Mining and Technology, Xuzhou 221116, China; ts22190076a31@cumt.edu.cn (J.H.); ds24190053p32@cumt.edu.cn (R.C.)

**Keywords:** underground monitoring interface, interface layout, visual search, eye tracking, usability

## Abstract

In underground mining operations, intelligent electronic monitoring and control systems have gradually become essential tools for practitioners to obtain operational information. Because underground mines are characterized by low-light environments that differ substantially from above-ground natural lighting, screen-related factors can strongly affect visual search tasks. It is therefore important to examine how interface layout influences visual search efficiency under low-illuminance mining conditions. This study utilized eye-tracking sensing technology to evaluate user performance within a simulated underground electronic monitoring and control system. Interface layout and ambient illuminance were set as experimental variables to investigate their effects on users’ search efficiency, quantitative eye-movement metrics, and usability satisfaction. The results showed that interface layout and ambient illuminance had significant main effects on task completion time, fixation count, saccade count, and subjective usability score. Among the tested layouts, the Three-Column Layout (THCL) showed the most favorable performance in task completion time, fixation count, and subjective usability score, while visual search efficiency was generally higher under the 50-lx condition.

## 1. Introduction

China is the world’s largest producer and consumer of coal [1], and safe coal mining is critical to energy security and economic stability [2]. Mine production is a complex process [3]. To support safety monitoring, mine production areas are usually equipped with extensive monitoring devices, which generate and display large amounts of operational information. During search tasks, users often need to identify or monitor multiple targets simultaneously rather than focus on a single target [4,5]. Compared with single-target visual search, multiple-target visual search increases cognitive load and reduces search efficiency [6,7].

Although mines rely on artificial lighting throughout the day, the underground lighting environment differs markedly from natural above-ground illumination. Areas shielded by equipment can be particularly dim. In visual information search tasks, screen brightness, contrast, interface layout, and related factors may strongly affect feature extraction and information processing. Previous studies have shown that, in low-brightness environments, screen brightness, text presentation, and other interface elements are influenced by luminance ratio, and appropriate adjustment can reduce visual fatigue during reading [8].

### 1.1. Influence of Interface Layout on Visual Search Efficiency

The human–machine interface is a core medium through which users and devices exchange information, feedback, and operational commands. Its layout directly affects information acquisition and task completion. From the perspective of visual search theory, interface layout determines not only how information is spatially organized, but also how users allocate attention, locate targets, and form search paths. Accordingly, interface layout should not be regarded merely as a matter of visual presentation. It is also a key design factor that influences information-processing efficiency and task performance.

A growing body of research has shown that layout design significantly affects visual search, usability, and task performance. He et al. [9] found that design features of train electronic guidance interfaces influenced visual search behavior, task performance, and subjective usability. Li et al. [10] reported that layout forms significantly affected the usability of data-visualization interfaces. Lin and Pan [11] further showed that information layout and auxiliary instruction display mode jointly influenced task efficiency and subjective experience. Zhou et al. [12], using eye-tracking and EEG data, demonstrated that interface design features affected both task performance and physiological responses.

Beyond task efficiency, layout differences also affect browsing paths, attentional processes, and user experience. Țichindelean et al. [13] showed that different forms of visual organization changed users’ browsing paths and usability perceptions in web interfaces. Szekely et al. [14] demonstrated that eye-tracking metrics can effectively reflect attentional processes and usability experience in mobile augmented reality tasks. Choi and Jang [15] found that combining usability testing with eye tracking provided a more comprehensive understanding of users’ interaction experience in patient monitoring systems, which is relevant to the present study because mine supervision systems are also monitoring-oriented and safety-critical. Aksu et al. [16] further showed that EEG and eye-tracking data can characterize changes in mental workload during task performance, providing methodological support for analyzing interface layout effects from the perspectives of cognitive load and information processing.

Overall, previous studies have provided substantial evidence that interface layout affects visual search efficiency, attentional allocation, and subjective evaluation. However, most related research has focused on web interfaces, data-visualization interfaces, mobile applications, augmented reality systems, or general monitoring interfaces. Research on mine supervision interfaces, which involve hierarchical information structures, strong task orientation, and safety-critical attributes, remains limited. Moreover, the influence of layout has not been sufficiently interpreted within a clear theoretical framework. From the perspective of visual search theory, layout affects how users distribute attention, segment information, and form search paths [6,7]. From the perspective of cognitive load, clearer and more structured layouts may reduce unnecessary processing demands, whereas less organized layouts may increase search burden and confirmation cost. Therefore, studying mine supervision interfaces is important not only for empirical comparison, but also for explaining how layout organization affects search efficiency and cognitive processing in safety-critical tasks.

### 1.2. Reading Behavior in Dark Environments

In addition to interface layout, ambient illuminance is another important factor affecting visual search efficiency and interface-use experience. From an information-processing perspective, users’ visual performance under low-illuminance conditions depends not only on interface content, but also on ambient light level, screen brightness, display luminance, and display mode. Therefore, interface layout in low-light contexts should be discussed in relation to the surrounding lighting environment.

Previous studies have shown that the interaction between low illuminance and display parameters directly affects visual fatigue and visual performance. Yao et al. [17] found that visual recognition efficiency for handheld infrared thermometer interfaces varied significantly under low ambient illuminance, suggesting that dim environments can directly affect how efficiently users perceive screen-based information. Zhou et al. [18] reported that the optimum display luminance of LCD screens used at night varied with ambient illuminance, indicating that visual performance depends strongly on the coordination between screen output and environmental lighting. Huang et al. [19] showed that ambient light significantly influenced users’ visual comfort when viewing tablet displays. Sun et al. [20] further demonstrated that eye-tracking features can effectively characterize eye-fatigue changes during electronic screen use, suggesting that eye-movement metrics reflect not only attentional allocation but also visual load under low-illuminance conditions. Sengsoon and Intaruk [21] showed that light mode and dark mode have different effects on visual fatigue and visual comfort. Na et al. [22] proposed an adaptive luminance-difference model between text and background for smartphone displays and showed that it improved reading speed, preference, and psychological comfort. Similarly, Tian et al. [23] found, using eye-tracking and EEG data, that screen brightness and color paradigms in nighttime low-light environments significantly affected visual fatigue.

Taken together, these studies suggest that visual performance in dark environments is shaped not only by ambient illuminance itself, but also by the interaction between display characteristics and users’ visual processing. In other words, low-illuminance conditions may affect both visual comfort and the efficiency with which interface information is perceived and processed.

Overall, previous studies have demonstrated that interface layout influences visual search efficiency and that low-illuminance environments affect visual comfort and reading behavior. However, these two lines of research have largely developed separately. Relatively few studies have examined how interface layout and ambient illuminance jointly influence visual search efficiency within the same experimental framework, particularly in safety-critical industrial contexts. Existing studies have also focused mainly on web interfaces, mobile devices, or general display environments, whereas research on mine supervision interfaces remains limited. From an information-processing perspective, ambient illuminance may influence not only visual comfort, but also the efficiency with which interface information is perceived, encoded, and processed [18,19,20,21,22,23,24]. This suggests that layout and illuminance should be understood as interrelated design conditions that jointly shape users’ search performance, eye-movement behavior, and subjective usability.

Accordingly, the research gap addressed in this study is both empirical and theoretical. Although previous studies have separately examined interface layout and low-illuminance conditions, relatively few have integrated these factors within a unified framework to explain how they jointly affect visual search efficiency in hierarchical and safety-critical industrial interfaces. In particular, existing research has not sufficiently connected visual search theory, cognitive load theory, and information-processing perspectives to explain the mechanisms through which layout and illuminance influence search performance, eye-movement behavior, and subjective usability in mine supervision interfaces.

To address this gap, the present study develops an integrated mechanism linking perceptual input, attentional guidance, and cognitive cost in screen-based hierarchical search. Ambient illuminance and interface layout jointly determine the quality of visual cues available to users: illuminance affects the clarity and stability of screen information, whereas layout determines whether this information can be organized into meaningful spatial groups. These cues guide visual search by shaping attention allocation, gaze transitions, and the narrowing of search from category-level areas to target-level items. When visual cues are clear and spatially organized, users can reduce irrelevant inspection, repeated comparison, and target-confirmation effort; when cues are weak or poorly organized, search uncertainty and cognitive-load demands increase. Therefore, visual search efficiency is understood as the outcome of a continuous mechanism in which perceptual input quality supports attentional guidance, and attentional guidance further determines cognitive cost and task performance.

Based on this framework, the present study investigates the effects of different layout modes and illuminance conditions on users’ visual search performance, visual behavior, and subjective usability in a mine monitoring system interface. By integrating behavioral, eye-movement, and subjective measures, this study aims not only to provide empirical evidence for interface optimization, but also to offer a more theory-informed explanation of how interface layout and ambient illuminance shape visual search efficiency under low-illuminance conditions.

## 2. Materials and Methods

### 2.1. Participants

A total of 32 participants were recruited from China University of Mining and Technology to take part in the visual-search experiment and the post-task usability evaluation. All participants had an educational background related to mining, safety, or related fields, and all had normal or corrected-to-normal vision. Participants were selected using stratified systematic sampling according to user experience level. Specifically, an eligible participant pool was first established based on the inclusion criteria. Participants were then stratified into high-, medium-, and low-experience groups according to their prior exposure to mine-related systems, monitoring tasks, and hierarchical interface operations. Within each stratum, participants were selected at a fixed sampling interval after a randomly determined starting point until the planned sample size for that stratum was reached.

The experience groups were defined based on task familiarity and prior exposure to mine-related systems. High-experience participants had participated in mine-related projects, internships, training, or similar monitoring-system tasks and were relatively familiar with mine supervision systems, hierarchical information structures, and target-search operations. Medium-experience participants had received relevant coursework, training, or project-based exposure related to mining, safety supervision, or intelligent mine systems, and had a basic understanding of mine supervision tasks, but had limited direct operational experience with mine supervision interfaces. Low-experience participants had a related educational background and basic knowledge of mining or safety contexts, but had little or no direct experience with mine supervision systems, monitoring interfaces, or similar target-search tasks.

Based on these criteria, the final sample included high-experience participants (*n* = 8), medium-experience participants (*n* = 16), and low-experience participants (*n* = 8), following an approximate allocation ratio of 1:2:1. The medium-experience group was assigned a larger proportion because it was considered the primary target-user group of the simulated mine supervision interface. In practical use, such systems are generally not intended mainly for complete novices or exclusively for highly experienced operators. Instead, they are more commonly used by users with relevant knowledge, training, or task familiarity, but without extensive practical operation experience. Therefore, this allocation was intended to better reflect the expected composition of potential users. The experience classification was used to describe and control the participant composition, rather than as an independent variable for inferential comparison.

The sample size was considered appropriate for the exploratory laboratory-based nature of this study and for the adopted 3 × 5 full-factorial within-subjects design. Each participant completed all 15 experimental conditions generated by crossing three illuminance levels with five interface layout modes. Thus, each layout–illuminance condition included data from the same 32 participants, allowing each participant to serve as their own control. This repeated-measures structure helped reduce the influence of inter-individual variability and improve the sensitivity of comparisons among layout and illuminance conditions.

Before the formal analysis, all eye-tracking records were checked for calibration quality, task compliance, and data completeness. All 32 participants completed the experiment and the post-task questionnaire successfully, and all datasets met the quality requirements. Therefore, no participants were excluded from the final analysis. The final analytical sample comprised 32 participants, including 18 men and 14 women, aged 22–26 years (*M* = 23.00, *SD* = 1.26).

Participation was voluntary, and written informed consent was obtained from all participants before the experiment. As the participants were students rather than professional mine operators, this sample was selected to maintain experimental control, ensure consistent task understanding, and isolate the effects of layout and illuminance in this early-stage laboratory study. Therefore, caution is needed when generalizing the findings to actual operational settings. This limitation is acknowledged here and further discussed in the Conclusions section.

### 2.2. Experimental Materials

**Figure 1:**
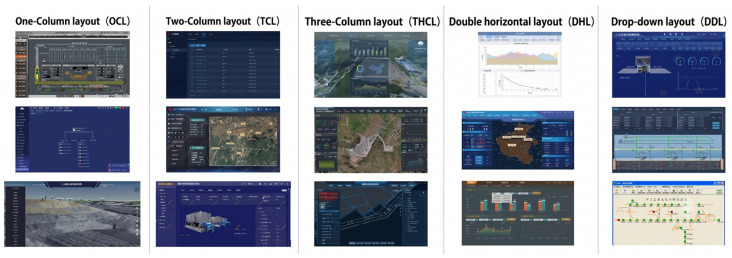
Classification of mine supervision system interfaces.

**Figure 2:**
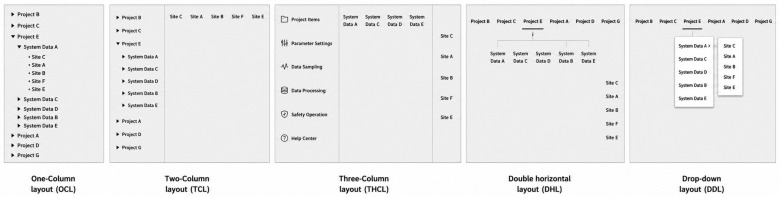
Experimental interface diagrams for the mine supervision system.

Based on preliminary investigation, coal mine supervision and monitoring system pages were divided into five categories, as shown in Figure 1. According to Na et al. [22], in dark environments (50 lx and below), a page with 85% background brightness (white) and 15% text brightness (black) provides favorable reading efficiency and comfort. All layouts in the present experiment used a three-level directory hierarchy. To prevent alphabetical order from affecting experimental accuracy, the order of headings at each level was randomized. As shown in Figure 2, the interfaces were grouped into five layout modes, presented from left to right as follows: One-Column Layout (OCL), Two-Column Layout (TCL), Three-Column Layout (THCL), Double Horizontal Layout (DHL), and Drop-down Layout (DDL).

To isolate interface layout as the core independent variable, the experimental interfaces were intentionally simplified. Non-structural visual cues, such as color coding, icons, and decorative graphic elements, were removed or minimized, and all layouts were presented with the same hierarchical depth and basic visual style. This design reduced potential confounding effects from salient visual cues unrelated to spatial organization and allowed observed differences in search performance to be more directly attributed to layout structure. However, this simplification also reduces ecological realism compared with operational mine supervision systems. Therefore, the present findings should be interpreted as evidence from a controlled layout-focused simulation rather than as a full reproduction of real-world mining interfaces.

### 2.3. Experimental Equipment and Procedures

#### 2.3.1. Experimental Equipment and Setup

Eye movements were recorded using a Tobii Pro X3-120 eye tracker (Tobii Pro, Beijing, China) eye-tracking device and the ErgoLAB platform for human–machine–environment synchronization. The stimuli were displayed on a 23-inch monitor with a resolution of 1920 × 1080 pixels. The viewing distance was set and maintained at 55 cm. Before measurement, each participant adapted to the room lighting conditions for 5 min and then completed screen calibration. According to the Chinese standard GB 50215-2015, Code for Design of Coal Mine Industry [25], the lighting standard for underground work should range from 10 lx to 100 lx and can be classified according to different working and operating areas as follows:

For underground roadways and operating areas, illuminance should be no lower than 50 lx to ensure that workers can clearly perceive the surrounding environment and avoid accidents. In loading and unloading areas, illuminance should be no lower than 100 lx to ensure safe operation. For safety passages and emergency exits, illuminance should be no lower than 20 lx to ensure rapid and safe evacuation in emergencies. Therefore, three environmental illuminance levels, namely 20 lx, 50 lx, and 100 lx, were used to simulate dark underground mining environments. According to the safety technical requirements of GB 7957-2023 for coal mine lamps [26], LED light sources should be used for mine lamps, and the correlated color temperature of the light source should be greater than 5000 K.

To simulate underground working conditions, the experiment was conducted in a laboratory darkroom after 8:00 p.m. Ambient illuminance was controlled using a JINBEI EF Panel 12 intelligent LED table lamp (Shanghai Jinbei Photographic Equipment Industry Co., Ltd., Shanghai, China), and the color temperature was maintained at 5000 K. According to the method for measuring environmental and surface brightness proposed by the National Ergonomics Standardization Technical Committee in 2009 [23], ambient illuminance in the darkroom was measured at the desktop level. Horizontal illuminance was measured at four points arranged in a quadrilateral configuration, and the average value was calculated. Display luminance was measured at five positions, including four points within a 4 cm × 4 cm area around the screen and one point at the center of the display. The average luminance value was then obtained. A SNAKOL SK-8200 (Shenzhen Snakol Electronics Co., Ltd., Shenzhen, Guangdong Province, China) illuminance and color-temperature meter was used to measure ambient illuminance and color temperature.

#### 2.3.2. Experimental Procedure

Before the experiment, participants read the experimental instructions and completed practice trials to familiarize themselves with the procedure. They were then informed of the target search tasks and pressed any key to begin the experiment.

**Figure 3:**
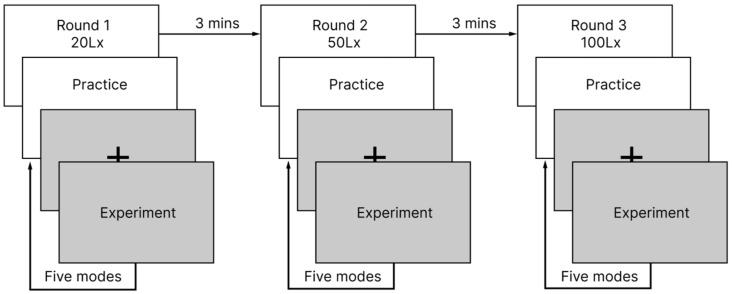
Experimental process.

The experiment adopted a 3 × 5 full-factorial within-subjects design with two independent variables: (1) environmental illuminance, with three levels (20 lx, 50 lx, and 100 lx), and (2) interface layout mode, with five levels: One-Column Layout (OCL), Two-Column Layout (TCL), Three-Column Layout (THCL), Drop-down Layout (DDL), and Double Horizontal Layout (DHL). Crossing these two variables yielded 15 experimental conditions, and each participant completed the task under all conditions. Due to laboratory environmental calibration and darkroom adaptation constraints, the three environmental illuminance conditions were administered in a fixed progressive order (20 lx, followed by 50 lx, and concluding with 100 lx), as structurally illustrated in Figure 3. To minimize potential confounding carry-over effects at the layout level, the five interface layout modes were fully randomized within each illuminance block for each participant.

At the beginning of each trial, a fixation cross (“+”) was presented at the center of the screen for 1 s to standardize the participant’s initial gaze position. Three representative search tasks were used in the experiment: “Engineering Project”, “System Data”, and “First Scene”. These terms represented three target-entry categories in the simulated mine supervision interface: an engineering-project information entry, a system-data entry, and a scene-monitoring entry. They were selected to represent typical navigation targets within the three-level hierarchical directory structure. To minimize the potential influence of task memory, a task-label randomization strategy was adopted. Specifically, each task name was appended with a unique sequence of random English letters for differentiation, and the order of these letter sequences was generated using the built-in randomization function in Microsoft Excel before each experiment, thereby randomizing the task execution order for each participant.

Participants performed the corresponding search tasks without a time limit, and task completion time was recorded automatically when each task was completed. The experimental procedure is illustrated in Figure 3. After the eye-tracking experiment, participants completed a usability evaluation questionnaire. All experimental materials were presented in Chinese because all participants were native Chinese speakers.

Before the formal experiment, participants received brief training on the basic characteristics of the interface layouts and completed practice trials to familiarize themselves with the task procedure. After the eye-tracking task, participants completed a usability questionnaire. A seven-point Likert scale was adopted, following Finstad [24], who suggested that seven-point ratings can provide greater sensitivity than five-point ratings. The questionnaire items were adapted from the Post-Study System Usability Questionnaire (PSSUQ), and selected items from the dimensions of system quality, information quality, and interface quality were retained to fit the present study context. Because all participants were native Chinese speakers, both the interface stimuli and the questionnaire were presented in Chinese.

### 2.4. Data Processing and Analysis

Eye-movement data were processed using ErgoLAB 3.0 Pro eye-tracking analysis software. Because the target options were randomized across trials, Areas of Interest (AOIs) were dynamically defined as the clickable hotspots corresponding to the target option in each task, with a uniform size of 200 × 40 pixels. AOI dwell time was defined as the total duration for which the participant’s gaze remained within the target AOI during a trial, reflecting the amount of visual attention allocated to the target region.

For each participant under each experimental condition, the following measures were recorded: task completion time, accuracy rate, fixation count, saccade count, AOI dwell time, and subjective usability score. Task completion time was defined as the interval between the presentation of the directory-information page and the participant’s mouse click on the target option, reflecting total search duration and overall search efficiency.

After preprocessing, the data were exported in TSV format and imported into IBM SPSS Statistics 27.0 for statistical analysis. Given the 3 × 5 within-subjects design, repeated-measures ANOVAs were conducted for each dependent variable, with interface layout (five levels: OCL, TCL, THCL, DDL, and DHL) and environmental illuminance (three levels: 20 lx, 50 lx, and 100 lx) as within-subject factors. The main effects of layout and illuminance, as well as their interaction effects, were examined. Partial eta squared (ηp^2^) was reported as the effect size for omnibus tests. When significant main effects were observed, Bonferroni-adjusted pairwise comparisons were performed. Descriptive statistics are reported as means, standard deviations, standard errors, and 95% confidence intervals.

## 3. Results

All data met the assumptions for parametric testing. This section reports the main inferential results using F and *p* values in the text, while detailed descriptive statistics and pairwise comparison values are presented in the corresponding tables.

### 3.1. Overall Effects of Interface Layout and Illuminance

As summarized in Table 1, interface layout and ambient illuminance showed significant main effects on most efficiency-related, eye-movement, and subjective indicators, including fixation count, saccade count, task completion time, subjective rating, and AOI dwell time. The interaction effects were generally not significant, indicating that the relative differences among layout modes were relatively stable across the three illuminance conditions. Compared with these indicators, accuracy was less informative for explaining search efficiency because it showed no significant illuminance or interaction effect, although its layout effect reached significance with a smaller effect size. Overall, these results indicate that layout and illuminance mainly influenced search efficiency, visual behavior, and subjective experience, rather than producing broad changes in task success.

As detailed in Table 2, Table 3, Table 4, Table 5 and Table 6, the Bonferroni comparisons further clarified the direction of these effects. Across illuminance levels, 50 lx generally supported faster search and better subjective experience than the other illuminance conditions. Across layout modes, THCL was associated with a more favorable search-efficiency profile than less structured alternatives, especially DDL. This pattern provides a basis for further discussing the role of layout structure in visual search in the following sections.

### 3.2. Layout Differences Under Each Illuminance Condition

**Figure 4:**
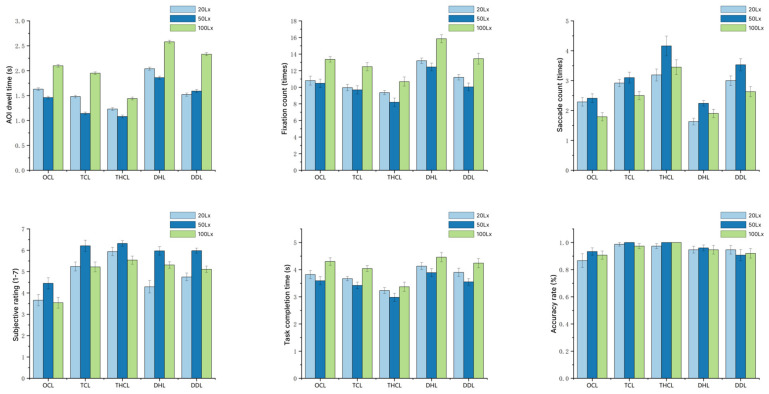
Mean comparisons of all measures across layouts under different illuminance levels.

As shown in Figure 4, the layout-wise comparisons across 20 lx, 50 lx, and 100 lx showed a generally consistent pattern. THCL presented the most favorable overall profile across the three illuminance levels, whereas DDL tended to show the weakest efficiency-related performance. This indicates that the favorable performance of THCL was observed across different illuminance conditions, rather than being limited to a single lighting level.

As shown in Table 7, under 20 lx, THCL showed a relatively favorable pattern in task completion time, fixation count, AOI dwell time, and subjective rating, whereas DDL showed a less efficient profile on most measures. This result suggests that, even under relatively low ambient illuminance, clear layout differences could still be observed in the participants’ search performance and visual behavior.

As shown in Table 8, under 50 lx, THCL showed the most favorable overall profile among the layout conditions, and TCL also performed relatively well on several indicators. Compared with OCL, DDL, and DHL, these two layouts showed more favorable patterns in efficiency-related and eye-movement measures, with THCL maintaining a slight overall advantage.

As shown in Table 9, under 100 lx, several efficiency-related and eye-movement indicators showed a less favorable pattern than under 50 lx. Nevertheless, THCL still retained its relative advantage over the other layouts, whereas DDL remained the relatively less favorable layout on several objective measures. This result further indicates that the relative ranking of layout modes was generally consistent across the tested illuminance conditions.

### 3.3. Illuminance Differences Across Layouts

**Figure 5:**
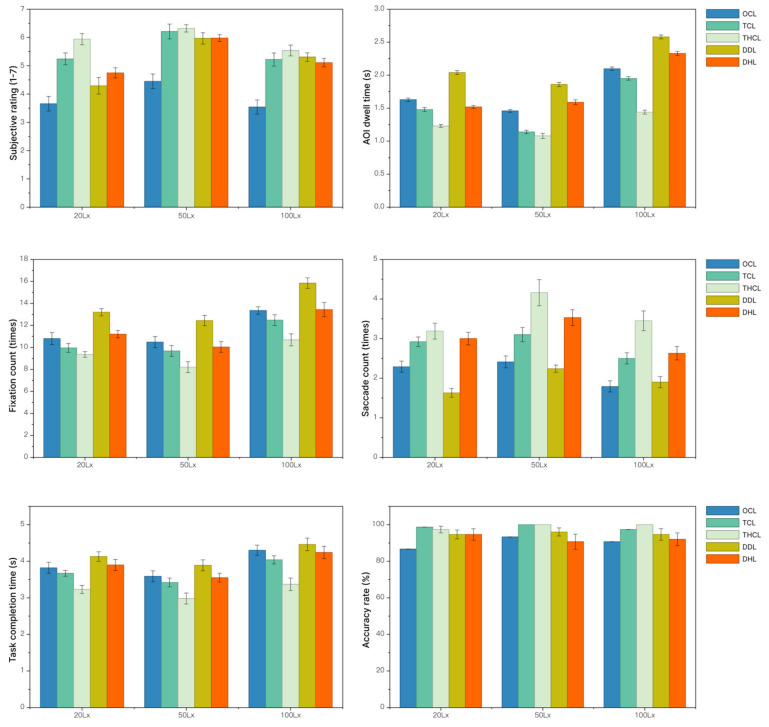
Mean comparisons of all measures across illuminance levels for different layouts.

As shown in Figure 5, the comparison across illuminance levels indicates that 50 lx generally produced the most favorable performance pattern across layouts, whereas 100 lx showed a less favorable pattern on several efficiency-related indicators. Across layouts, 50 lx was generally associated with fewer fixations, shorter AOI dwell time, shorter task completion time, and higher subjective ratings than 100 lx. However, because the interaction effects were generally not significant, illuminance should be interpreted as a broad contextual factor that influenced overall search performance, rather than as a condition that fundamentally changed the relative ranking of layout modes.

Taken together, the results indicate that THCL and 50 lx produced the most favorable performance pattern under the present laboratory-simulated task conditions. THCL showed a consistent advantage across different illuminance levels, while 50 lx was generally associated with better efficiency-related, eye-movement, and subjective indicators than 100 lx. The theoretical and practical implications of these findings are discussed in the following section.

## 4. Discussion

The present study shows that interface layout had a clear effect on visual search performance in the simulated mine-monitoring task. Although accuracy varied across layouts, its overall effect size was relatively small compared with those of the efficiency-related and eye-movement measures. Therefore, task completion time remained the primary indicator for interpreting search efficiency. Under the present experimental conditions, THCL showed the most favorable performance pattern, particularly in terms of shorter task completion time, fewer fixations, and shorter AOI dwell time, indicating that it supported hierarchical information retrieval more effectively than the other layout modes.

### 4.1. Performance on Search Tasks

The present study indicates that interface layout influenced visual search efficiency in the simulated mine-monitoring interface. Accuracy was not used as the primary basis for interpreting search efficiency because, although the layout effect reached significance, its effect size was smaller than those of the efficiency-related and eye-movement indicators, and accuracy did not show significant illuminance or interaction effects. This indicates that participants maintained a comparable level of task success under different experimental conditions; therefore, accuracy could not be used to explain differences in search efficiency. Accordingly, task completion time was adopted as the primary indicator of search efficiency, because it directly reflected the time required for users to locate and confirm target information. Under the present laboratory-simulated conditions, THCL showed the most favorable overall performance pattern, suggesting that it provided relatively effective support for hierarchical information retrieval.

The favorable performance of THCL can be interpreted through this combined explanatory framework. In the present hierarchical retrieval task, users needed to identify a relevant information category before locating the target item within that category. THCL may have supported this process by presenting information as clearer and more organized visual cues. Its three-column structure separated information into distinct spatial regions, which may have made category-level information easier to perceive, encode, and compare. These organized cues could then guide attention more effectively, helping users narrow the search space from broader information groups to specific target items. As a result, users may have required less irrelevant inspections, repeated comparisons, and prolonged target confirmation.

This pattern may be understood in relation to the task characteristics of the present interface. The mine supervision interface represented a hierarchical retrieval task, in which users first identified a relevant category and then located the target within that category. In such tasks, layout efficiency is likely to depend less on the absolute number of visible regions than on whether the interface provides clear grouping, stable structural cues, and a manageable search space. Classical menu-search studies have shown that hierarchical retrieval performance is shaped by breadth, depth, and response cost [27], while more recent research on graphical user interfaces suggests that visual search often follows a “Guess–Scan–Confirm” process guided by structural expectations [28]. From this perspective, layout may influence not only where users look, but also how efficiently they narrow the search space and confirm the target.

The relative advantage of THCL may therefore be explained by its clearer hierarchical partitioning. Hornof [29] showed that labeled hierarchical displays are easier to search than unlabeled ones, and Hornof and Halverson [30,31] further demonstrated that users tend to search group labels before narrowing attention to the target group. Brumby and Zhuang [32] likewise found that visual grouping is more effective when it is aligned with the underlying semantic structure. These studies support the mechanism proposed in the present study: effective layout does not merely increase the amount of visible information, but transforms interface content into usable visual cues that guide attention and reduce search uncertainty. In THCL, the spatial separation of information regions may have strengthened the mapping between category-level structure and target-level localization, thereby helping users move from global category identification to local target confirmation more efficiently. In the present study, THCL appears to have provided a clearer mapping between interface regions and task-relevant information categories, which may have reduced the effective search space and facilitated faster target localization.

Its relative advantage may also be related to spatial consistency and lower confirmation cost. Prior studies suggest that users gradually learn where to look through repeated interaction [33], that spatially consistent layouts can support location memory and reduce search time [34], and that more ordered layouts may reduce perceived interface complexity [35]. In the present study, THCL may have provided more stable spatial cues than the other layouts, thereby reducing repeated inspection during target confirmation. This interpretation is consistent with the observed pattern of fewer fixations, shorter completion time, and shorter AOI dwell time.

TCL also performed relatively well overall, suggesting that it retained some of the benefits of hierarchical grouping. By contrast, OCL may have increased the burden of sequential scanning because its single-column structure tends to lengthen the search path [27,28]. DDL showed a less favorable pattern on several objective measures. Although expandable structures can reduce the amount of simultaneously visible information, prior studies suggest that hiding information may also shift part of the burden to expansion, path tracking, and context maintenance [36,37]. This may help explain the relatively weaker performance of DDL in the present task. DHL also appeared less well matched to the current retrieval logic, as its region arrangement may have increased transitions between areas and repeated local checking. Overall, the present findings suggest that, for hierarchical retrieval in the current mine-monitoring task, layouts with clearer semantic grouping, more stable spatial organization, and moderate visible complexity may better support efficient search. This interpretation should be limited to the present laboratory-simulated interface and task setting rather than generalized to all interface types.

### 4.2. Differences in Visual Behavior

This interpretation is consistent with previous work on hierarchical search and eye-movement behavior. Hornof and Halverson [30] reported that fixation count is closely related to search time and that more efficient hierarchical interfaces are generally associated with fewer and shorter fixations. In the present study, the lower fixation count and shorter AOI dwell time observed in THCL may indicate that users required less repeated inspection and less local processing to confirm the target. AOI dwell time is especially informative because it reflects the time required for target confirmation within the relevant region, rather than only the overall amount of scanning. A shorter AOI dwell time in THCL therefore suggests a lower confirmation burden and more direct target identification.

The eye-movement findings further clarify how the proposed mechanism operated during the search process. Fixation count and saccade count reflect how users distribute attention and move between information regions, whereas AOI dwell time reflects the amount of local processing required for target confirmation. Therefore, these eye-movement indicators connect visual search behavior with cognitive-load demands. In THCL, fewer fixations and shorter AOI dwell time suggest that users encountered less uncertainty when identifying the relevant information region and required less effort when confirming the target. The relatively higher saccade count should therefore be interpreted together with the reduced fixation-related measures: it may indicate more efficient transitions among structured regions rather than inefficient visual wandering. This pattern is consistent with a search process in which organized visual cues support faster global scanning and less effortful local verification.

The relatively higher saccade count observed in THCL should not be interpreted as an indicator of lower efficiency or elevated search costs. As underscored in classical eye-tracking methodologies, a single eye-movement metric can be functionally ambiguous and must be evaluated within a joint multi-metric framework rather than in isolation [38,39]. Visual disorientation or high interface processing costs typically trigger a concurrent surge in both saccade and fixation counts, as users repeatedly re-scan poorly organized regions to resolve uncertainty [38,39]. In this context, the specific performance profile of THCL—characterized by an increased saccade count coupled with fewer fixations (Table 2) and the shortest task completion time (Table 4)—firmly corroborates a structured and purposeful visual scanning strategy rather than erratic visual wandering. As suggested by Over et al. [40], visual search often follows a coarse-to-fine process, in which rapid global scanning is followed by more detailed local inspection. Vasilyev and Zhou et al. [41,42] further argued that fixations and saccades should be interpreted jointly rather than independently. Therefore, the combination of fewer fixations, shorter AOI dwell time, and relatively more saccades in THCL demonstrates that users were able to shift gaze efficiently between structured information regions and confirm the target more rapidly once it was reached, achieving an optimal balance between rapid global transition and low-effort local verification.

By contrast, TCL showed slightly longer fixation-related measures than THCL, suggesting somewhat greater local processing demands, although it still performed relatively well overall. DHL generally produced more fixations and fewer saccades than THCL, which may indicate less efficient transitions between information regions and greater reliance on repeated local inspection. DDL showed the least favorable pattern on several objective measures, including fixation count, task completion time, and AOI dwell time, suggesting that its structure may have imposed a higher search burden. Overall, these results indicate that THCL and, to a lesser extent, TCL are better suited to hierarchical information search, whereas layouts with weaker structural guidance may require more effortful visual processing.

### 4.3. Subjective Usability

The subjective usability results generally followed the same direction as the behavioral and eye-movement findings. Layouts associated with fewer fixations, shorter task completion times, and shorter AOI dwell times tended to receive more favorable ratings, suggesting that users were sensitive not only to task success but also to the efficiency and fluency of the search process itself. This pattern is consistent with Chevalier [43], who showed that interfaces associated with greater search burden tend to receive less favorable usability evaluations. In the present study, THCL and TCL generally received more favorable ratings, whereas OCL tended to receive the lowest ratings.

However, the subjective results should be interpreted with caution. Although THCL showed the strongest overall objective performance, its subjective rating did not differ significantly from that of TCL or DDL in the post hoc comparisons, while it was significantly higher than that of DHL. This suggests that subjective usability was broadly consistent with the objective results, but not perfectly equivalent to them. In other words, users may perceive several layouts as acceptable even when objective efficiency differs across layouts. Therefore, the value of THCL lies not only in its favorable subjective evaluation, but also in its stronger and more consistent objective efficiency. This convergence between subjective and objective evidence strengthens the practical relevance of THCL for mine supervision interfaces intended to support repeated and prolonged use.

### 4.4. Ambient Illuminance

With respect to ambient illuminance, the present results indicate that the 50-lx condition provided the most favorable overall performance pattern, whereas the 100-lx condition generally showed the least favorable pattern. Across layouts, 50 lx was associated with fewer fixations, shorter task completion time, shorter AOI dwell time, and higher subjective ratings than 100 lx, and it also outperformed 20 lx on several overall measures. These findings tentatively suggest that, under the present laboratory-simulated screen-based task conditions, a moderate low-illuminance level may support visual search more effectively than a brighter ambient environment. However, this interpretation must be treated with caution. Because the illuminance blocks were administered in a fixed chronological sequence, the observed performance peak at 50 lx and the subsequent decline at 100 lx might be partially modulated by cumulative practice effects or onset fatigue rather than environmental illuminance alone.

Therefore, the strength of direct causal inferences regarding the illuminance main effect should be softened until fully verified by counterbalanced designs. Unlike interface layout, which mainly affects the spatial organization of visual cues, ambient illuminance mainly affects the perceptual quality of these cues. Under the 50-lx condition, the balance between ambient light and screen output may have made interface elements easier to distinguish and category-level information easier to encode. This more stable perceptual input could support visual search by helping users rely on clearer spatial and categorical cues during global scanning and local target confirmation. In contrast, the less favorable pattern under 100 lx may be associated with stronger screen–environment luminance competition, which could increase visual discrimination effort and target-confirmation cost. This interpretation is broadly consistent with Zhou et al. [18] and with previous studies showing that visual performance in dark environments depends on the coordination between ambient illumination and display-related conditions rather than on ambient light level alone.

This interpretation should remain cautious because the present study did not directly measure glare, luminance contrast, or visual strain. Nevertheless, the observed increases in fixation count, AOI dwell time, and task completion time under 100 lx suggest that this condition may have increased the effort required for visual discrimination and target confirmation. At the same time, although both 20 lx and 50 lx fell within the low-illuminance range relevant to underground settings, 50 lx showed a more consistently favorable pattern across efficiency, eye-movement, and subjective measures. Therefore, the present findings do not suggest that lower illuminance is always better; rather, they indicate that, under the specific laboratory simulation and stimulus conditions of this study, 50 lx provided a more suitable balance for visual information extraction and search performance than 100 lx and, on several measures, than 20 lx.

### 4.5. Theoretical and Practical Implications

The present findings provide implications for both HCI theory and interface design practice. From a theoretical perspective, this study extends existing HCI research on visual search and interface organization by showing that layout structure and ambient illuminance should be understood as interrelated design conditions rather than isolated variables. In particular, the results suggest that clearer hierarchical layouts may facilitate more efficient attentional guidance, reduce confirmation cost, and support more fluent visual search, whereas ambient illuminance may influence not only visual comfort but also the efficiency with which screen-based information is perceived and processed. In this sense, the present study contributes to the broader HCI literature by linking visual search behavior, cognitive processing, and usability evaluation in a safety-critical monitoring context under low-illuminance conditions.

From a practical perspective, the findings offer preliminary guidance for the design of real-world monitoring interfaces used in low-light environments. For screen-based monitoring tasks involving hierarchical information retrieval, layouts with clearer partitioning and stronger structural guidance, such as the three-column layout, may better support efficient target localization and reduce search burden. By contrast, layouts with weaker hierarchical guidance may increase local confirmation cost and reduce search efficiency. In addition, under the present laboratory-simulated conditions, the 50-lx environment provided a more favorable balance of search efficiency, eye-movement performance, and subjective usability than the 100-lx condition. This suggests that the design of monitoring workstations for low-light environments should consider not only the interface layout itself, but also the balance between ambient illumination and efficient visual information extraction. These implications should be interpreted cautiously because the present study was conducted in a laboratory-simulated setting with simplified interfaces; nevertheless, they provide an initial basis for future design optimization in safety-critical monitoring systems.

## 5. Conclusions

In the context of intelligent mining, this study examined how interface layout and ambient illumination affect search performance, eye-movement behavior, and subjective usability in a laboratory-simulated mine monitoring task. The results showed that interface layout had a clear influence on search efficiency: THCL was associated with the shortest task completion time, the fewest fixations, and a relatively higher saccade count, whereas DDL showed a less favorable pattern on several objective measures. These findings suggest that a more structured hierarchical layout may reduce visual search burden and support more efficient information processing. Subjective usability generally varied in the same direction as the behavioral and eye-movement measures, with higher ratings observed in conditions associated with shorter task completion times, fewer fixations, and higher saccade counts. With respect to illumination, the 50-lx condition was associated with shorter completion times, fewer fixations, and a higher saccade count than 100 lx, although differences between 20 lx and 50 lx were small for some eye-movement and subjective measures. Under the specific laboratory simulation and stimulus conditions of this study, 50 lx tended to perform better than 100 lx and, on some measures, slightly better than 20 lx. Overall, these findings provide preliminary evidence that interface layout and ambient illumination jointly influence user performance in low-light screen-based tasks, while also offering an initial basis for future research and interface design under more realistic mining conditions.

This study has several limitations that should be addressed in future work. First, the experiment was conducted in a laboratory-simulated setting and therefore could not fully reproduce the complexity of real mining environments. Future studies should validate the present findings in more realistic operational contexts. Second, although the within-subjects design allowed each participant to complete all 15 experimental conditions and helped reduce inter-individual variability, the overall sample size remained relatively limited. This limitation was mainly related to the controlled eye-tracking procedure, the requirement to maintain consistent low-illuminance laboratory conditions, and the practical difficulty of recruiting a large number of participants with mine-related operational experience within the current experimental setting. In addition, the participants were university students with mining- or safety-related educational backgrounds rather than professional mine operators, which limits the direct ecological generalizability of the current findings. Future studies should expand the participant pool through multi-site and multi-source recruitment to incorporate actual mine-monitoring operators across different age groups and diverse levels of professional work experience. Where feasible, future research should adopt a rigorous stratified systematic sampling strategy—first classifying participants by their age cohorts and years of frontline operational experience, and then selecting individuals at fixed sampling intervals within each stratum. This approach will significantly mitigate selection bias, yield a more representative and balanced sample, and substantially enhance the generalizability of the empirical results to real-world industrial monitoring environments. Third, the present study focused mainly on interface layout and ambient illuminance, while other environmental factors relevant to mining work, such as noise, stress, fatigue, and time pressure, were not considered. Fourth, a critical methodological limitation is that the three ambient illuminance conditions followed a fixed sequential order (20 lx → 50 lx → 100 lx) without counterbalancing via a Latin square design. Consequently, potential practice effects (explaining the peak performance at 50 lx) or fatigue effects (explaining the efficiency drop at 100 lx) cannot be entirely disentangled from the authentic main effect of illuminance. Finally, the tested interfaces were relatively simplified. Future research could examine more complex and realistic interface designs, including richer task flows, denser information displays, and more dynamic interaction scenarios, to further assess the applicability of the current findings.

## Figures and Tables

**Table 1:** Within-subjects tests for all outcome measures.

Measure	Source	Type III Sum of Squares	df	Mean Square	F	*p*	Partial η^2^
Fixation Count	Illuminance	607.637	2	303.819	51.236	0.000	0.623
	Layout	777.211	4	194.303	32.895	0.000	0.515
	Illuminance × Layout	21.429	8	2.679	0.534	0.830	0.017
Saccade Count	Illuminance	27.181	2	13.590	26.453	0.000	0.460
	Layout	138.274	4	34.568	44.168	0.000	0.588
	Illuminance × Layout	10.273	8	1.284	0.774	0.634	0.024
Task Completion Time	Illuminance	22.310	2	11.155	20.282	0.000	0.395
	Layout	38.956	4	9.739	16.139	0.000	0.342
	Illuminance × Layout	1.090	8	0.136	0.356	0.942	0.011
Accuracy Rate	Illuminance	164.037	2	82.019	0.725	0.490	0.023
	Layout	4425.669	4	1106.417	2.937	0.024	0.087
	Illuminance × Layout	841.963	8	105.245	1.086	0.374	0.034
Subjective Rating	Illuminance	65.284	2	32.642	23.886	0.000	0.435
	Layout	187.646	4	46.911	36.882	0.000	0.543
	Illuminance × Layout	48.931	8	6.116	1.517	0.152	0.047
AOI Dwell Time	Illuminance	14.173	2	7.087	66.315	0.000	0.681
	Layout	34.415	4	8.604	257.855	0.000	0.893
	Illuminance × Layout	1.092	8	0.136	1.452	0.187	0.045

**Table 2:** Bonferroni post hoc comparisons for fixation count.

Factor	Pair	Mean Difference	SE	*p*	95% Confidence Interval	
					Lower	Upper
Illuminance	20 lx × 50 lx	0.736	0.201	0.004	0.219	1.253
	20 lx × 100 lx	−2.256	0.355	0.000	−3.170	−1.342
	50 lx × 100 lx	−2.992	0.344	0.000	−3.876	−2.108
Layout	OCL × TCL	0.840	0.433	0.644	−0.499	2.179
	OCL × THCL	2.133	0.420	0.000	0.836	3.431
	OCL × DDL	−2.280	0.412	0.000	−3.552	−1.008
	OCL × DHL	−0.013	0.434	1.000	−1.355	1.328
	TCL × THCL	1.293	0.375	0.021	0.133	2.453
	TCL × DDL	−3.120	0.410	0.000	−4.386	−1.854
	TCL × DHL	−0.853	0.322	0.139	−1.847	0.140
	THCL × DDL	−4.413	0.274	0.000	−5.259	−3.567
	THCL × DHL	−2.147	0.405	0.000	−3.399	−0.895
	DDL × DHL	2.267	0.450	0.000	0.877	3.656

**Table 3:** Bonferroni post hoc comparisons for saccade count.

Factor	Pair	Mean Difference	SE	*p*	95% Confidence Interval	
					Lower	Upper
Illuminance	20 lx × 50 lx	−0.482	0.103	0.000	−0.748	−0.216
	20 lx × 100 lx	0.149	0.063	0.078	−0.013	0.311
	50 lx × 100 lx	0.631	0.100	0.000	0.373	0.888
Layout	OCL × TCL	−0.681	0.105	0.000	−1.006	−0.356
	OCL × THCL	−1.438	0.170	0.000	−1.964	−0.912
	OCL × DDL	0.236	0.132	0.857	−0.171	0.644
	OCL × DHL	−0.892	0.122	0.000	−1.270	−0.514
	TCL × THCL	−0.758	0.164	0.001	−1.266	−0.249
	TCL × DDL	0.917	0.104	0.000	0.595	1.239
	TCL × DHL	−0.211	0.106	0.580	−0.539	0.117
	THCL × DDL	1.675	0.203	0.000	1.048	2.301
	THCL × DHL	0.546	0.187	0.075	−0.032	1.125
	DDL × DHL	−1.128	0.105	0.000	−1.454	−0.802

**Table 4:** Bonferroni post hoc comparisons for task completion time.

Factor	Pair	Mean Difference	SE	*p*	95% Confidence Interval	
					Lower	Upper
Illuminance	20 lx × 50 lx	0.267	0.073	0.004	0.079	0.455
	20 lx × 100 lx	−0.329	0.097	0.007	−0.579	−0.079
	50 lx × 100 lx	−0.596	0.108	0.000	−0.874	−0.319
Layout	OCL × TCL	0.195	0.095	0.505	−0.098	0.487
	OCL × THCL	0.708 ×	0.158	0.002	0.221	1.196
	OCL × DDL	−0.256	0.101	0.182	−0.568	0.056
	OCL × DHL	0.007	0.145	1.000	−0.442	0.457
	TCL × THCL	0.513 ×	0.097	0.000	0.214	0.812
	TCL × DDL	−0.451 ×	0.104	0.002	−0.771	−0.130
	TCL × DHL	−0.188	0.115	1.000	−0.544	0.168
	THCL × DDL	−0.964 ×	0.149	0.000	−1.425	−0.503
	THCL × DHL	−0.701 ×	0.133	0.000	−1.111	−0.291
	DDL × DHL	0.263	0.150	0.921	−0.200	0.727

**Table 5:** Bonferroni post hoc comparisons for subjective rating.

Factor	Pair	Mean Difference	SE	*p*	95% Confidence Interval	
					Lower	Upper
Illuminance	20 lx × 50 lx	−1.010	0.146	0.000	−1.386	−0.634
	20 lx × 100 lx	−0.370	0.160	0.089	−0.781	0.042
	50 lx × 100 lx	0.640	0.137	0.000	0.288	0.992
Layout	OCL × TCL	−1.673	0.195	0.000	−2.274	−1.071
	OCL × THCL	−2.049	0.225	0.000	−2.744	−1.355
	OCL × DDL	−1.640	0.249	0.000	−2.409	−0.871
	OCL × DHL	−1.397	0.181	0.000	−1.957	−0.836
	TCL × THCL	−0.377	0.162	0.292	−0.879	0.125
	TCL × DDL	0.033	0.197	1.000	−0.577	0.643
	TCL × DHL	0.276	0.139	0.585	−0.153	0.705
	THCL × DDL	0.409	0.182	0.340	−0.153	0.972
	THCL × DHL	0.653	0.119	0.000	0.284	1.021
	DDL × DHL	0.243	0.155	1.000	−0.236	0.722

**Table 6:** Bonferroni post hoc comparisons for AOI dwell time.

Factor	Pair	Mean Difference	SE	*p*	95% Confidence Interval	
					Lower	Upper
Illuminance	20 lx × 50 lx	0.356 ×	0.033	0.000	0.271	0.441
	20 lx × 100 lx	−0.096	0.052	0.235	−0.230	0.038
	50 lx × 100 lx	−0.452 ×	0.037	0.000	−0.546	−0.358
Layout	OCL × TCL	0.205 ×	0.027	0.000	0.122	0.289
	OCL × THCL	0.479 ×	0.031	0.000	0.383	0.574
	OCL × DDL	−0.431 ×	0.030	0.000	−0.525	−0.337
	OCL × DHL	−0.085	0.028	0.058	−0.171	0.002
	TCL × THCL	0.273 ×	0.030	0.000	0.181	0.366
	TCL × DDL	−0.637 ×	0.027	0.000	−0.720	−0.553
	TCL × DHL	−0.290 ×	0.031	0.000	−0.387	−0.193
	THCL × DDL	−0.910 ×	0.020	0.000	−0.973	−0.847
	THCL × DHL	−0.563 ×	0.038	0.000	−0.679	−0.447
	DDL × DHL	0.347 ×	0.033	0.000	0.246	0.448

**Table 7:** Descriptive statistics at 20 lx.

Measure	Layout	Mean	SD	SE	95% CI	
					Lower	Bound
Fixation Count	OCL	10.80	2.68	0.474	9.70	11.91
	TCL	9.96	2.01	0.355	9.13	10.79
	THCL	9.36	1.29	0.228	8.83	9.89
	DDL	13.20	1.66	0.293	12.52	13.88
	DHL	11.20	1.66	0.293	10.52	11.88
Saccade Count	OCL	2.29	0.71	0.126	1.99	2.58
	TCL	2.92	0.62	0.110	2.66	3.18
	THCL	3.19	0.99	0.175	2.78	3.60
	DDL	1.63	0.53	0.094	1.41	1.85
	DHL	3.00	0.80	0.141	2.67	3.33
Task Completion Time	OCL	3.82	0.74	0.131	3.52	4.13
	TCL	3.67	0.41	0.072	3.50	3.84
	THCL	3.23	0.56	0.099	3.00	3.47
	DDL	4.13	0.65	0.115	3.86	4.40
	DHL	3.90	0.76	0.134	3.59	4.21
Subjective Rating	OCL	3.66	1.31	0.232	3.12	4.20
	TCL	5.24	1.05	0.186	4.80	5.67
	THCL	5.94	1.02	0.180	5.52	6.36
	DDL	4.29	1.45	0.256	3.69	4.89
	DHL	4.75	0.89	0.157	4.38	5.12
AOI Dwell Time	OCL	1.629	0.141	0.025	1.576	1.682
	TCL	1.480	0.124	0.022	1.424	1.536
	THCL	1.230	0.108	0.019	1.169	1.291
	DDL	2.040	0.181	0.032	1.978	2.102
	DHL	1.520	0.136	0.024	1.456	1.584

**Table 8:** Descriptive statistics at 50 lx.

Source	Layout	Mean	SD	SE	95% CI	
					Lower	Bound
Fixation Count	OCL	10.48	2.52	0.445	9.44	11.52
	TCL	9.68	2.44	0.431	8.67	10.69
	THCL	8.20	2.50	0.442	7.17	9.23
	DDL	12.44	2.33	0.412	11.48	13.40
	DHL	10.04	2.42	0.428	9.04	11.04
Saccade Count	OCL	2.41	0.75	0.133	2.09	2.72
	TCL	3.10	0.90	0.159	2.73	3.47
	THCL	4.16	1.66	0.293	3.47	4.85
	DDL	2.24	0.46	0.081	2.05	2.43
	DHL	3.53	0.98	0.173	3.12	3.93
Task Completion Time	OCL	3.59	0.73	0.129	3.29	3.89
	TCL	3.42	0.58	0.103	3.18	3.66
	THCL	2.98	0.75	0.133	2.67	3.29
	DDL	3.89	0.73	0.129	3.58	4.19
	DHL	3.55	0.62	0.110	3.29	3.80
Subjective Rating	OCL	4.45	1.32	0.233	3.90	5.00
	TCL	6.21	1.31	0.232	5.67	6.75
	THCL	6.32	0.65	0.115	6.05	6.58
	DDL	5.97	1.02	0.180	5.55	6.39
	DHL	5.98	0.59	0.104	5.74	6.22
AOI Dwell Time	OCL	1.459	0.119	0.021	1.406	1.512
	TCL	1.140	0.096	0.017	1.084	1.196
	THCL	1.080	0.091	0.016	1.019	1.141
	DDL	1.860	0.153	0.027	1.798	1.922
	DHL	1.590	0.130	0.023	1.526	1.654

**Table 9:** Descriptive statistics at 100 lx.

Source	Layout	Mean	SD	SE	95% CI	
					Lower	Bound
Fixation Count	OCL	13.36	1.63	0.288	12.69	14.03
	TCL	12.48	2.45	0.433	11.47	13.49
	THCL	10.68	2.76	0.488	9.54	11.82
	DDL	15.84	2.39	0.422	14.85	16.83
	DHL	13.44	3.27	0.578	12.09	14.79
Saccade Count	OCL	1.79	0.68	0.120	1.51	2.07
	TCL	2.50	0.71	0.126	2.21	2.80
	THCL	3.45	1.24	0.219	2.94	3.96
	DDL	1.90	0.68	0.120	1.62	2.19
	DHL	2.63	0.83	0.147	2.29	2.98
Task Completion Time	OCL	4.30	0.72	0.127	4.00	4.59
	TCL	4.04	0.56	0.099	3.81	4.27
	THCL	3.37	0.86	0.152	3.02	3.73
	DDL	4.46	0.84	0.148	4.11	4.80
	DHL	4.24	0.87	0.154	3.88	4.60
Subjective Rating	OCL	3.54	1.26	0.223	3.02	4.06
	TCL	5.22	1.16	0.205	4.75	5.70
	THCL	5.54	0.95	0.168	5.15	5.93
	DDL	5.31	0.76	0.134	5.00	5.62
	DHL	5.11	0.73	0.129	4.81	5.41
AOI Dwell Time	OCL	2.099	0.128	0.023	2.046	2.152
	TCL	1.950	0.136	0.024	1.894	2.006
	THCL	1.440	0.148	0.026	1.379	1.501
	DDL	2.580	0.151	0.027	2.518	2.642
	DHL	2.330	0.156	0.028	2.266	2.394

## Data Availability

The raw eye-tracking data generated during the current study, including fixation count, saccade count, and task completion time, together with the subjective usability questionnaire data, are available from the corresponding author (Wei Zong, zongwei@cumt.edu.cn) upon reasonable request. The interface layout materials used in the experiment are not publicly available because of the research design of the mine supervision system, but they can be provided by the authors for research purposes.
